# Predictive values of sex and tumour site for survival benefit from 5FU in colorectal cancer

**DOI:** 10.1038/sj.bjc.6600279

**Published:** 2002-05-06

**Authors:** B Iacopetta

**Affiliations:** Department of Surgery, University of Western Australia, Nedlands 6009, Australia

## Abstract

*British Journal of Cancer* (2002) **86**, 1524–1525. DOI: 10.1038/sj/bjc/6600279 www.bjcancer.com

© 2002 Cancer Research UK

## Sir

The report by Taal *et al* (2001), on behalf of the Netherlands Adjuvant Colorectal Cancer Project (NACCP), provides further evidence of the benefits of 5-fluorouracil (5FU)-based regimes for the adjuvant treatment of colorectal cancer (CRC). This multi-centred trial entered a total of 1029 patients into control (surgery alone) and treatment (surgery+12 months 5FU/Levamisole) arms. With a median of almost 5 years follow-up, the results show survival improvements of 8% and 12% for stage II and III patients, respectively. Also of interest are the survival benefits observed in the tumour site, gender and age subgroups (Figure 5). Most of the improved survival is attributable to colon cancer patients, while female patients appear to derive approximately twice as much benefit as males. Although not cited by Taal *et al* (2001), we have reported similar site and sex differences in the survival benefit from 5FU/Leucovorin in a non-randomized, retrospective series of Dukes' C CRC patients (Elsaleh *et al*, 2000). We were led to these observations by studies on the prognostic significance of the microsatellite instability (MSI+) phenotype. By comparing the survival of patients treated with or without chemotherapy, we noted an excellent survival benefit for MSI+ cases. Knowing that MSI+ tumours are found almost exclusively in the proximal colon (Thibodeau *et al*, 1993) and that sex-related differences had been reported for this phenotype (Breivik *et al*, 1997), we were prompted to examine various site and sex subgroups for the survival benefit from 5FU.

In contrast to what is stated in the editorial accompanying the NACCP study (Maughan, 2001), we found that the MSI+ group could not account for the entire survival benefit from 5FU (Elsaleh *et al*, 2000). Although the frequency of MSI+ tumours in sporadic CRC is similar to the degree of observed benefit (approximately 10%), we also found that MSI-patients showed significantly improved survival with 5FU treatment. The molecular features associated with response to 5FU remain to be fully characterized, however both preclinical (Bunz *et al*, 1999) and clinical (Ahnen *et al*, 1998; Elsaleh *et al*, 2001) studies have demonstrated a requirement for normal *TP53*. It is therefore interesting to note that ‘responsive’ MSI+ tumours nearly always have normal *TP53*, whereas ‘non-responsive’ distal tumours are frequently *TP53* mutant (Breivik *et al*, 1997; Elsaleh *et al*, 2001). Another insight from the pioneering work of Breivik *et al* (1997), which also has relevance to the NACCP findings, is that MSI+ tumours are most frequent in younger male and older female CRC patients. It may only be coincidental, but the youngest (<60 years) and oldest (>65 years) patient subgroups in the study by Taal *et al* (2001) derive more survival benefit than the 60–65 year age group (Figure 5).

What are the implications of the NACCP study and what role, if any, for molecular predictive factors in the management of CRC? Clinicians and patients who were previously reticent about the benefits of 5FU treatment for stage II disease should now feel more confident about its use, particularly for females and those with proximal cancers. In contrast, there appears little survival benefit to be gained from the use of 5FU in males with rectal cancers. These patients should be encouraged to enter trials for experimental drugs such as oxaliplatin and irinotecan, perhaps even as first line treatments. At this stage there seems little justification for the routine use of any of the known molecular predictive markers. MSI+ is highly specific but lacks sensitivity for the detection of all 5FU responsive tumours (Elsaleh *et al*, 2000). *TP53* is not sufficiently specific, since some patient groups with a mutation, most notably females, still show an apparent survival benefit from 5FU (Elsaleh *et al*, 2001). Future studies may find that other molecular features such as DNA methylation or certain gene expression patterns revealed by microarray analysis can provide sensitive and specific predictive information for the survival benefit from 5FU. Until such time as these markers are incorporated into prospective clinical trials in order to evaluate their true worth, the predictive values of tumour site and sex should not be neglected. Both factors are likely to be determinants of CRC subgroups that have different molecular features and therefore different biological and clinical properties, including the response to various adjuvant therapies.
